# Prevalence of Carcinoma in Appendectomy Specimens for Patients Presenting With Acute Appendicitis: A Single-Center Study

**DOI:** 10.7759/cureus.19611

**Published:** 2021-11-15

**Authors:** Omotara Lesi, Sarah-Jane Walton, Nikhil Nanjappa Ballanamada Appaiah, Noreen Rasheed, Jayasiri Dahanayaka, Philip Ideawor, Abdalla Saad Abdalla Al-Zawi

**Affiliations:** 1 General and Colorectal Surgery, Basildon and Thurrock University Hospital, Mid and South Essex NHS Foundation Trust, Basildon, GBR; 2 Radiology, Basildon and Thurrock University Hospital, Mid and South Essex NHS Foundation Trust, Basildon, GBR; 3 General and Breast Surgery, Basildon and Thurrock University Hospital, Mid and South Essex NHS Foundation Trust, Basildon, GBR; 4 Pathology, Basildon and Thurrock University Hospital, Mid and South Essex NHS Foundation Trust, Basildon, GBR; 5 General and Breast Surgery, Anglia Ruskin University, Chelmsford, GBR

**Keywords:** colonoscopy, carcinoid, adenocarcinoma, appendicectomy, acute appendicitis

## Abstract

Introduction

Acute appendicitis is the most common general surgical emergency globally. Its etiology includes the presence of luminal obstruction by faecoliths, lymphoid hyperplasia, impacted stool, and rarely by appendiceal or caecal cancer. Malignancy related to acute appendicitis is usually seen in the older age group.

Aim

To identify the subset rate of patients operated for acute appendicitis who have appendiceal carcinoma and analyze the outcome of their post-operative management.

Material and methods

A retrospective study of a cohort of 529 patients aged > 40 diagnosed with acute appendicitis with subsequent appendectomy in the period between 1 January 2014 and 31 December 2019 at Basildon and Thurrock University Hospital, Essex, United Kingdom was conducted. We analyzed the clinical data of the cohort including demographic information, diagnosis, pre-operative imaging, histological diagnosis as well as post-operative management where indicated.

Results

The median age of patients was 54.5 years (range 40-92). The male to female ratio in the appendicectomy cohort was 1:1.1. About 45% were aged 40-49 years, 24.8% were aged 50-59 and 30.2% were ≥60 years. Post-operative histology revealed acute appendicitis in 82.4% of the group. In 11% of the patients, the histology revealed the presence of other benign pathology as mucocele of the appendix, acute diverticulitis, follicular hyperplasia, and fibrous obliteration. The diagnosis of appendicular malignancy was seen in 1.9%.

Conclusion

Incidental appendiceal cancers in the resected specimens after acute appendicitis are rare but may be associated with a poor prognosis. It is recommended to consider such diagnosis in particular when dealing with acute appendicitis in older patients with longer symptom history, and in presence of peri-appendicular mass.

## Introduction

Acute appendicitis is one of the most common causes of acute abdomen in adults with an estimated lifetime risk of 7-8% [1]. Also, it is recognized as is the most frequent general surgical emergency in the world with around 50,000 appendicectomies performed yearly in the UK [2]. The highest incidence occurs in the second and third decades of life, with the pathology less common in both extremes of life [3]. The etiology includes the presence of luminal obstruction by fecaliths, lymphoid hyperplasia, impacted stool, parasitic infestation, and rarely by an appendiceal or caecal cancer [3, 4]. The obstruction of the lumen can lead to increased intramural pressure thereby affecting venous and lymphatic outflow. This will subsequently lead to impaired vascular and lymphatic flow with attendant ischemia. The inflammatory process can result in perforation, abscess formation as well as generalized peritonitis [5]. Luminal obstruction by neoplasms is usually seen in the elderly, this relationship was first reported by Dr. Shears in 1906 [6].

The aim of this study was to determine the incidence of appendicular cancer in patients over 40 years who had undergone appendicectomy as well as assess their further management outcome.

## Materials and methods

This retrospective study analyzed data of patients ≥ 40 years who underwent appendicectomy in the period between 1 January 2014 and 31 December 2019 at Basildon and Thurrock University Hospital, Essex, United Kingdom. Patients were collated from the hospital’s clinical portal record. Data collected included demographic data (gender, age, etc), pre-operative imaging, initial diagnosis, intra-operative findings and histological diagnosis, post-operative management. 

The inclusion criteria included patients with a diagnosis of acute appendicitis and no other acute abdominal pathology identified as well as patients with proven appendiceal cancers after surgery. Those excluded were patients with acute abdomen of unknown cause. 

## Results

A total of 529 appendicectomies were performed during the study period. The M:F ratio in the cohort was 1:1.1. The median age of patients was 54.5 years (range 40-92 years). Around 45%, or 238 patients were aged 40-49 years, 131 patients (24.8%) were aged 50-59 and 160 patients (30.2%) were 60 years and above (Table 1).

**Table 1:** Age distribution of all patients who underwent appendicectomy

Age Range (Years)	N (%)
40-49	238 (45%)
50-59	131 (24.8%)
60-69	90 (17%)
70-79	56 (10.6%)
80-89	13 (2.5%)
≥ 90	1 (0.2%)

Pre-operative investigations were done in the majority of patients: 487 patients (92%) had pre-operative CT scans, 20 patients (3.8%) had abdominal ultrasound scans, two patients had magnetic resonance imaging (MRI) while 20 patients did not have any pre-operative investigations.

Histology diagnosis revealed the presence of acute appendicitis in 82.4% of patients. There was negative appendicectomy in 4.7% of the patients while there was an incidental finding of appendicular tumors in 10 patients (1.9%). In 11% of the patients, there was the presence of mucocele of the appendix, acute diverticulitis, follicular hyperplasia, and fibrous obliteration (Table 2). 

**Table 2:** The histopathology of the resected appendices in 529 patients

Appendicular pathology	N(%)	
Acute Appendicitis	436(82.4%)	
Other benign changes	58(11%)	
Normal	25(4.7%)	
Malignant tumours	10(1.9%)	
Neuro-endocrine tumours		6(1%)
Adenocarcinoma		4(0.8%)

The incidental appendicular malignancies included: neuroendocrine tumors, adenocarcinoma of the appendix, and mucinous adenocarcinoma of the appendix (Table 3).

**Table 3:** Malignant appendiceal tumors detected in 10 patients with pre-operative diagnosis of acute appendicitis AA: acute appendicitis; EMA: emergency appendicectomy; NET: neuroendocrine tumour (carcinoid); WD: well differentiated; PD: poorly differentiated; SFU: surgical follow-up

No.	Age	Sex	Pre-op CT	Operative procedure	Operative findings	Appendicular histology	T stage	Adjuvant treatment
01	45	F	AA	EMA	AA	Adenocarcinoma of appendix	T4	Referred to tertiary centre
02	46	F	AA	EMA	AA	AA, WD NET	T1	SFU
03	53	F	AA	EMA	Necrotic AA	AA with NET	T1	SFU
04	54	F	AA	EMA	AA, abscess	NET	T1	SFU
05	64	F	AA	EMA	AA, abscess	mucinous adenocarcinoma	T3	Right hemicolectomy
06	65	M	AA	EMA	AA, abscess	WD, NET	T1	SFU
07	67	M	AA	EMA	AA	PD mucinous adenocarcinoma	T4	Right hemicolectomy -declined
08	68	M	AA	EMA	AA	NET	T4 M1	Palliative
09	68	F	AA	EMA	AA, abscess	PD mucinous secreting adenocarcinoma	T4	Cytoreductive surgery and intra-peritoneal chemo
10	74	F	Perforated AA	EMA	AA, abscess	NET	T4	Right hemicolectomy

Post-operative bowel investigations were done in only 79 patients (14.9%) with abnormal findings seen in 29 patients (5.5%). Out of the 79 patients, only 19 patients (3.6%) had their post-operative investigations within three months of appendicectomy while the remaining 60 patients had theirs between six months to six years of the study period for varying reasons like change in bowel habits or through bowel cancer screening program.

Also, 26 patients (4.9%) had benign polyps detected during colonoscopies, another patient had high-grade dysplastic polyp while yet another person was diagnosed with caecal cancer. The patient with caecal cancer had an appendicectomy for CT-confirmed and histological diagnosis of acute appendicitis a year prior to cancer diagnosis. The following year after surgery, he was investigated for anemia and colonoscopy confirmed the presence of caecal cancer in the appendiceal orifice. He later had a right hemicolectomy and histology showed advanced colon cancer (pT4bN0M0). A second patient had a histology diagnosis of T3 appendiceal tumor after appendicectomy. Radical surgery was advised; however, the patient declined surgery and any luminal investigations within three months and CT staging was done five months later for abdominal symptoms which revealed a separate transverse colon tumor with metastases. The patient was sent for palliative care.

## Discussion

The first reported case of an appendiceal tumor was in 1882 [7], other studies have appeared in the literature since 1903 when Elting reported a review and case series [8]. Appendiceal tumors are rare, about 0.9%-1.4% of the tumors are seen during histology diagnosis of resected appendiceal specimens. The age-adjusted incidence is 0.12 cases per one million people per year [9], it is reported in both genders, however, some authors claim that the majority of patients were female [10, 11]. The most common primary appendiceal tumor is carcinoid tumor and it makes up 32-85% while adenocarcinomas (mucinous, signet ring, or non-mucinous) make up 4-20% of the tumors [12]. In our study, out of the 529 patients aged over 40 years who underwent appendicectomies, 10 patients had malignant appendiceal tumors with 60% of the tumors showing carcinoids while 40% were revealed to be adenocarcinomas in the histopathology. Appendiceal tumors are mostly located at the tip of the appendix with a maximal size of <1 cm in 60-80% of cases with a five-year survival of 83% for all stages [13].

**Figure 1:**
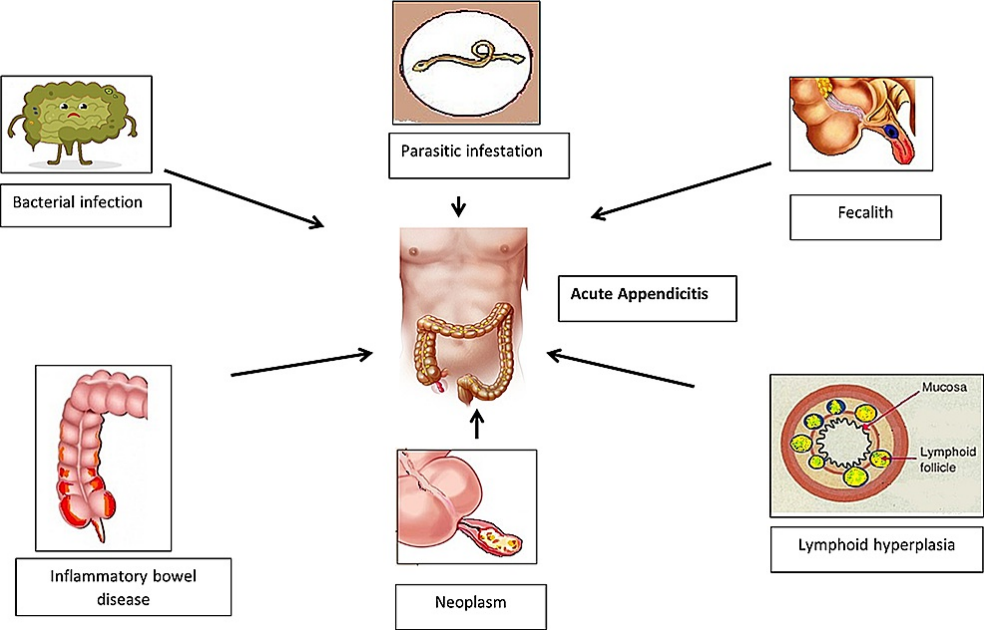
Causes of acute appendicitis Illustration by Dr Abdalla Saad Abdalla Al-Zawi.

In the majority of cases of appendiceal malignancy, patients present with symptoms of acute appendicitis or a palpable mass [14]. This was similar in our study with all the patients presenting with features of acute appendicitis [14]. Acute appendicitis is usually caused by fecaliths, lymphoid hyperplasia, impacted stool, parasitic infestation, and rarely by a neoplasm (see Figure 1). Patients could also present features of a perforated appendix if the neoplasm obstructs the lumen of the appendix [12]. Rarer features include the presence of pelvic mass, hydronephrosis, Crohn's disease, haematuria, anemia, vesico-appendiceal fistula, and caecal intussusception [5, 15, 16]. The presence of symptoms of carcinoid syndrome occurring in patients with carcinoid tumors is rare, and in usual circumstances, they indicate the presence of liver metastases. In patients with carcinoid tumors, levels of urinary 5-hydroxy-indoleacetic acid (5-HIAA), urinary and serum serotonin levels can be used to monitor the progression of the disease [9].

**Figure 2:**
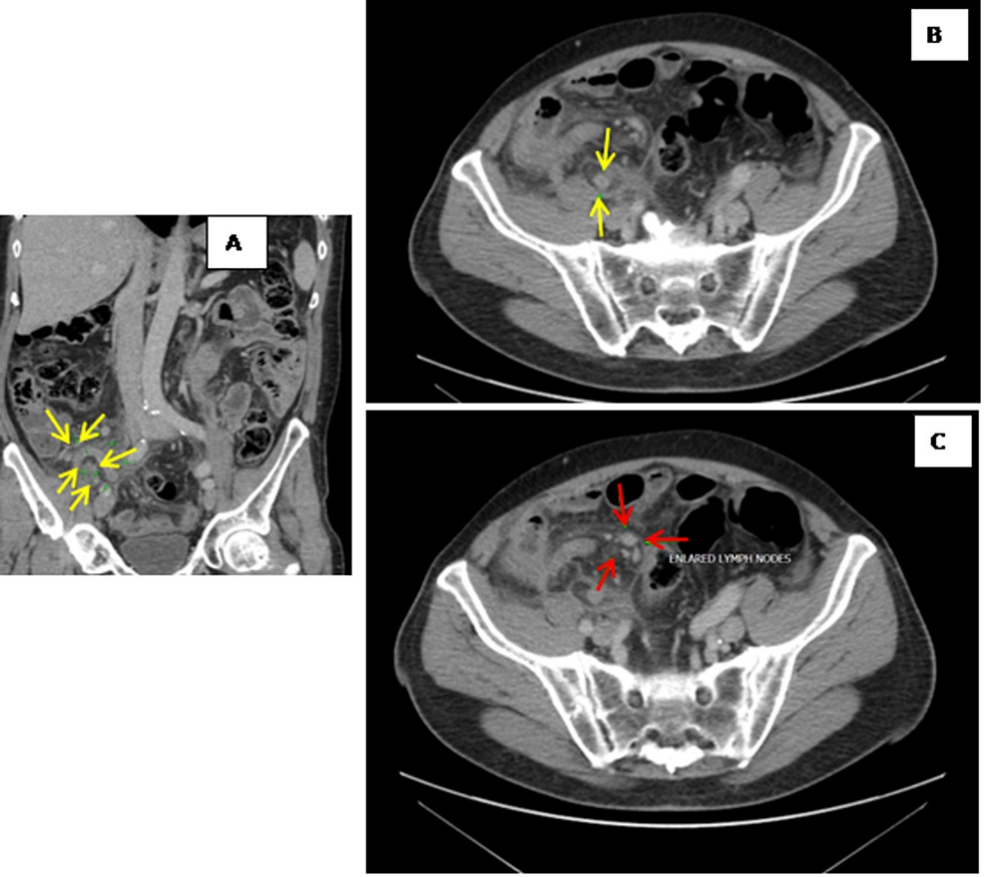
CT scan of abdomen and pelvis: (A) coronal view, (B) axial view, (C) axial view of pelvic CT scan showing evidence of acute appendicitis There is evidence of acute appendicitis with thickening of the appendix measuring 10 mm in diameter, and marked inflammatory stranding in the periappendiceal and pericaecal fat (A-B, yellow arrows). There is a periappendiceal fluid collection measuring 20 x 25 mm (A-B, yellow arrows). The appendix is seen extending into the right hemi-pelvis. Several enlarged mesenteric nodes were noted to be medial to the appendix (C), red arrows. The post-operative histology showed appendiceal cancer.

**Figure 3:**
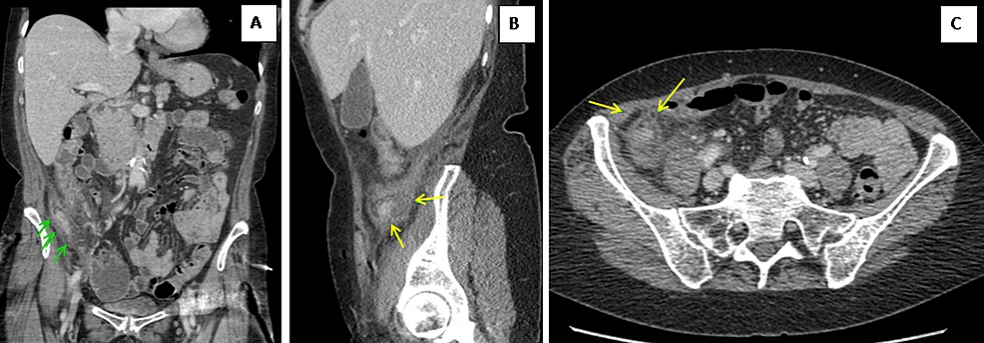
Contrast-enhanced CT images with coronal (A), sagittal (B) and axial (C) reformats demonstrate heterogeneous enhancement of dilated appendix (A, green arrows) with associated significant fat stranding and free fluid within the right para colic gutter (B-C, yellow arrows) Appearances were suggestive of acute appendicitis. The post-operative histology showed appendiceal cancer.

In this study, the diagnosis of appendiceal tumors was not made pre-operatively; this is also seen in the published studies [17]. The radiological findings of appendiceal carcinoids are limited due to the small size of the tumors and their location in the distal aspect of the appendix (Figures 2-3). Ultrasound scan findings include the presence of a hyperechoic round mass at the tip of the appendix where CT findings suggestive of carcinoid tumor include the presence of a focal, soft tissue mass with enhancement. On MRIs, they appear as T1 isointense and T2 isointense-hyperintense masses with contrast enhancement [18]. Adenocarcinoma on the other hand appears in the CT scan as a subtle infiltrating mass, as well as prominent inflammatory changes around the appendix as well as enlargement of the appendix CTS scan [19].

**Figure 4:**
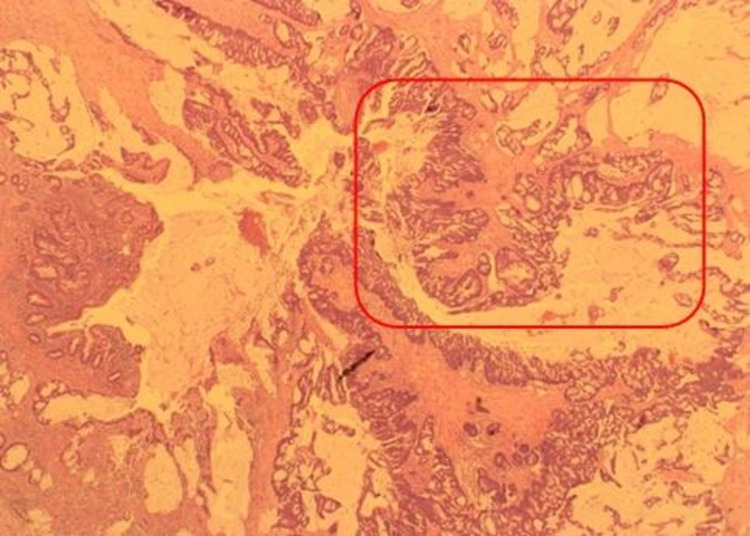
Appendix showing extensive infiltration of the wall by a mucinous adenocarcinoma (red box)

The International Classification of Diseases for Oncology (ICD-O), 2nd edition groups appendiceal tumors into five categories: colonic-type (non-mucinous) adenocarcinoma, mucinous adenocarcinoma, signet-ring cell carcinoma, goblet cell carcinoid/adenocarcinoid, and malignant carcinoid [20]. The non-mucinous adenocarcinoma occurs less frequently than the mucinous type (Figure 4) in which mucin is involved in more than 50% of the lesion [12, 21]. Our study shows that majority of the patients with adenocarcinoma had a mucinous type. Adenocarcinomas usually arise from an adenomatous polyp or serrated adenoma. The mucinous type usually causes myxoma peritonei and about half of patients with mucinous tumors will usually have transcoelomic spread and associated pseudomyxoma peritonei [22]. McCusker et al., in 2002, reported that showed a five-year survival rate of 44% for mucinous subtype, 52% for colonic subtype, and 20% for signet ring cell subtype [20].

Carcinoid tumors of the appendix could be classical carcinoid or goblet cell carcinoid tumors. Classical carcinoid tumors arise from neuroendocrine tissue in the primitive gastrointestinal tract [9], while goblet cell carcinoid arises from pluripotent intestinal epithelial crypt-base cells, it is characterized by dual neuroendocrine and mucinous differentiation [23]. Goblet cell carcinoid has pathologic features which are seen in both appendiceal carcinoid and colonic signet ring cell adenocarcinoma and it is noted that 20% of the tumors, also have a propensity for metastases to ovaries and peritoneum [24]. Our study did not distinguish between goblet cell carcinoid and classical carcinoid appendiceal tumors. The management of appendiceal tumors post appendectomies depends on the histological type, size, and location of the tumor. In patients with goblet cell carcinoids, a right hemicolectomy is usually performed after the initial appendicectomy, this is because the rate of metastases is high [25]. The completion surgery should be done within three months of the appendicectomy operation. Appendicectomy alone could be done in those with localized T1 tumors (<1cm). Patients with larger tumors, lesions locally advanced as in invasion of the caecum, serosa, or mesoappendix advised having right hemicolectomy. It is also recommended that women with this tumor should have bilateral salpingo-oophorectomy regardless of age. Those with peritoneal carcinomatosis may have multiple peritonectomies as well as intraperitoneal chemotherapy while patients with liver metastases [25].

All the patients in this study diagnosed with T1 carcinoid tumors had only appendicectomies except one of them who had a right hemicolectomy for a 10mm tumor invading the muscle and with Ki67 less than 10%. Adjuvant chemotherapy is recommended in those with lymph node involvement while those with intra-abdominal metastases require aggressive debulking surgery followed by adjuvant chemo-radiotherapy [26]. Hata et al. published a review in 2002, the paper reiterated that well-differentiated adenocarcinoma invading the submucosa or adenocarcinoma of any differentiation limited to the mucosa can be safely treated with appendicectomy [27]. On the other hand, adenocarcinoma with lymphatic/vascular invasion, poorly differentiated tumors, those with a massive invasion of the submucosa as well as advanced appendicular cancer are treated with secondary right hemicolectomy with lymph node excision. All the patients in this study with advanced disease were offered adjuvant treatment such as right hemicolectomy, cytoreductive surgery, or chemotherapy. Appendicular carcinoma exhibit a variable spectrum of biological behavior, the patient gender, and tumor pathomorphological are associated with OS (overall survival) in advanced cases of appendiceal carcinoma [10]. The published reports have shown that elevated pre-management tumor marker levels as CEA, CA 19-9 and CA125, to be associated with higher rates of disease recurrence and reduced survival after complete cytoreductive surgery. Other factors associated with poor prognoses include poor tumor differentiation and extension beyond the appendiceal mucosa [28]. The Ki-67 proliferative index which has been placed in prognosis prediction in some areas as breast cancer [29], claimed to be of no prognostic significance for some appendicular tumors as goblet cell carcinoid tumors [30].

## Conclusions

Fortunately, incidental appendiceal cancers in the resected specimens after acute appendicitis are rare, however may be associated with poor prognosis. It is recommended to consider such diagnosis in particular when dealing with acute appendicitis in older patients with longer symptom history, and in presence of periappendicular mass. There were some limitations to the study; small sample size and a short follow-up period. The patients reviewed were over 40 years and though the incidence is higher in older age groups, some younger patients with malignancy would have been not included in the study.
